# Supplementary material for: Aphid facultative symbionts confer no protection against the fungal entomopathogen Batkoa apiculata
Source: PLoS One. 2023 May 19;18(5):e0286095. doi: 10.1371/journal.pone.0286095 (PMC10198479; doi:10.1371/journal.pone.0286095)
Supplement: S1 Table — (DOCX) [file pone.0286095.s001.docx]

Table S1: PCR primers used for symbiont screening.

| Organism | Gene | Forward name | Forward Primer 5' to 3' | Reverse name | Reverse Primer 5' to 3' |
| --- | --- | --- | --- | --- | --- |
| *Hamiltonella defensa.* | 16S rRNA | 10F | AGTTTGATCATGGCTCAGATTG | T419R | AAATGGTATTSGCATTTATCG |
| *Regiella insecticola* | 16S rRNA | 10F | AGTTTGATCATGGCTCAGATTG | TO419R | GGTAACGTCAATCGATAAGCA |
| *Serratia symbiotica* | 16S rRNA | 16SA1 | AGAGTTTGATCMTGGCTCAG | 16S.S2R | TTTGAGTTCCCGACTTTATCG |
| *Fukatsuia symbiotica* | 16S rRNA | 20F | AGTTTGATCATGGCTCAGATTG | X420R | GCAACACTCTTTGCATTGCT |
| *Rickettsia* sp. | 16S rRNA | 16SA1 | AGAGTTTGATCMTGGCTCAG | 16S.Ri2R | TTTGAAAGCAATTCCGAGGT |
| *Spiroplasma* sp. | dnaA | SpDnaAF1 | ATTCTTCAGTAAAAATGCTTGGA | SpDnaAR1 | ACACATTTACTTCATGCTATTGA |
| *Rickettsiella* sp. | 16S rRNA | RCL16S-211F | GGGCCTTGCGCTCTAGGT | RCL16S-470R | TGGGTACCGTCACAGTAATCGA |
