# Supplementary material for: Aphid facultative symbionts confer no protection against the fungal entomopathogen Batkoa apiculata
Source: PLoS One. 2023 May 19;18(5):e0286095. doi: 10.1371/journal.pone.0286095 (PMC10198479; doi:10.1371/journal.pone.0286095)
Supplement: Attachment — Submitted filename: Review of PONE-D-23-00043 - Aphid facultative symbionts.pdf [file pone.0286095.s003.pdf]

Review of PONE-D-23-00043 - Aphid facultative symbionts confer no protection against the fungal entomopathogen *Batkoa apiculata* by Inchauregui, Tallapragada, and Parker

Comments for authors

I like the idea of the MS. Pea aphids have a number of microbial endosymbionts, some of which have protective functions. Here the authors focus on antifungal activities, and attempt to show that the mechanisms that protect the aphids against one common fungal pathogen may not protect against a closely related fungus. This is a limited objective, but potentially, an informative and consequential one.

I have two sets of reservations about the MS. In each case, I suspect that the authors may have the required information, but simply haven't yet provided it in the MS. Please note, my expertise does not extend to the more molecular methods used to identify pathogens. I cannot speak to that work.

Methodology

- Pea aphids have a variety of (mostly) vertically transmitted, microbial endosymbionts including some not mentioned here (e.g., *Buchnera aphidicola, Hamiltonella defensa* - *Buchnera* is unlikely to be defensive, but is an obligate symbiont, *Hamiltonella* has several roles.) The bacteria mediate a number of services to the aphids, and occur in various combinations which may interact and may be functionally and mechanistically important (see refs below). This makes me ask the following methodological questions:

1) How was the aphid stock 'cured' of *R. insecticola* (lines 108-110)? - in particular, which other endosymbionts were (likely) cured in the same process, and what endosymbionts (likely) remained in the stock?

2) Did you assess the effectiveness of the endosymbiont infection injections in establishing infections with the desired endosymbionts (lines 115-118)? Similarly, did you attempt to assess which other endosymbionts (if any) were harbored by the various lines produced?

3) Were there any procedural controls that assessed the effectiveness and uniformity of success of the methods used to infect the aphids with the fungi? I ask particularly because the strongly significant block effect (line 167), when the blocks were treated as fixed variables, suggests not just that repeated blocks differed, but that block 1 differed systematically from block 2. Unless, blocks 1 & 2 differed systematically as part of the experimental design, the fungal infection rates should not have differed strongly and significantly. If that effect is real, it should be presented and explained.

Without those clarifications, it is difficult to assess the meaning of Fig. 2.

Statistics

I agree that the fungal infection/protection data should be analyzed via a generalized liner model, but:

4) We need a better description of the model used. What type of GLM was used? The methods suggest that a logistic/binomial-family GLM was most appropriate, but the symmetric error bars on Fig. 2 and the use of the 'multcomp' package made me

suspect that a normal/gaussian GLM was used. With infection rates running as low as 10%, I suspect that the logistic GLM is much more defensible.

5) I was surprised that you chose to model the block effect as a fixed effect. Unless Block 1 really did differ systematically from Block 2 in the design of the experiment (e.g., Block 1 was done first and there was a time/duration/extinction effect), it seems more defensible to treat the blocks as random (blocking) effects in a mixed model (e.g., via the 'glmer' function in the lme4 package). This is particularly important when comparing the honest-to-goodness fixed effects (here the lines/symbionts) because the blocking effects, in classical theory, become part of the error variance for testing the fixed effects. The 'glmer' function handles this properly. - - I think we can ignore the 'shrinkage' effects commonly discussed in mixed model random effects because you don't get much shrinkage with only two blocks with nearly balanced sample sizes.
6) I have always been nervous about using the 'multcomp' package for logistic regressions when some of the effects are close to 0 or 1 (e.g., 0.1 Fig. 2). For GLM's, multcomp uses a likelihood ratio test that assumes the likelihood is symmetrically distributed around the MLE, which may well not be true in logistic regressions (with some extreme effects) because of the non-linear transformation of the linear result (the logit) to the probability value, and the hard bound on probabilities of infection at 0 and 1. One way to get around this is to use a Bayesian regression and directly test the 'Bayesian p values' comparing each line to the 'None' control.

Smaller points - Fig. 2

- I was confused by the 'Percent sporulated' label on the dependent axis. Isn't this just percent cadavers (line 137-138)?
- The confidence intervals for each line will be asymmetric in a logistic regression, so the '+/- 1 SE' error bars can be misleading. I'd suggest converting to confidence intervals.

References:

Oliver, K. M., Moran, N. A., & Hunter, M. S. (2005). Variation in resistance to parasitism in aphids is due to symbionts not host genotype. PNAS 102(36), 12795-12800.

Rock, D. I., et al.. (2018). Context-dependent vertical transmission shapes strong endosymbiont community structure in the pea aphid, *Acyrthosiphon pisum*. Molecular Ecology, 27(8), 2039-2056.

Smith, A. H., et al. (2015). Patterns, causes and consequences of defensive microbiome dynamics across multiple scales. Molecular Ecology, 24(5), 1135-1149.
