# Supplementary material for: Aphid facultative symbionts confer no protection against the fungal entomopathogen Batkoa apiculata
Source: PLoS One. 2023 May 19;18(5):e0286095. doi: 10.1371/journal.pone.0286095 (PMC10198479; doi:10.1371/journal.pone.0286095)
Supplement: Attachment — Submitted filename: Response_letter.docx [file pone.0286095.s004.docx]

Reviewer #1: It has been well known that some facultative symbiotic bacteria protect aphids from infection by certain fungal strains. However it is not clear whether this protection is universal in term of lines of aphids, strains of symbiotic bacteria and pathogenic fungi. In this manuscript, the authors present solid evidences showing such symbiont-mediated protection does not exist to infection against Batkoa apiculate. These results will lead further study on ecological and molecular mechanisms of symbiont-mediated protection in aphids.

**Thanks**

Reviewer #2:  I like the idea of the MS. Pea aphids have a number of microbial endosymbionts, some of which have protective functions. Here the authors focus on antifungal activities, and attempt to show that the mechanisms that protect the aphids against one common fungal pathogen may not protect against a closely related fungus. This is a limited objective, but potentially, an informative and consequential one.

I have two sets of reservations about the MS. In each case, I suspect that the authors may have the required information, but simply haven’t yet provided it in the MS. Please note, my expertise does not extend to the more molecular methods used to identify pathogens. I cannot speak to that work.

Methodology
- Pea aphids have a variety of (mostly) vertically transmitted, microbial endosymbionts including some not mentioned here (e.g., *Buchnera aphidicola*, *Hamiltonella defensa* - *Buchnera* is unlikely to be defensive, but is an obligate symbiont, *Hamiltonella* has several roles.) The bacteria mediate a number of services to the aphids, and occur in various combinations which may interact and may be functionally and mechanistically important (see refs below). This makes me ask the following methodological questions:

1) How was the aphid stock ‘cured’ of *R. insecticola* (lines 108-110)? - in particular, which other endosymbionts were (likely) cured in the same process, and what endosymbionts (likely) remained in the stock?

**The aphid line used here, (LSR1) was originally collected with *Regiella insecticola* and then subsequently cleared using an antibiotic—the antibiotic (Ampicillin) does not affect *Buchnera aphidicola.* This line is used frequently by researchers in the field; the line does not contain any other secondary symbionts. Thanks for seeking this clarification—we’ve updated the text in the revised submission (lines 109-111).**

2) Did you assess the effectiveness of the endosymbiont infection injections in establishing infections with the desired endosymbionts (lines 115-118)? Similarly, did you attempt to assess which other endosymbionts (if any) were harbored by the various lines produced?

**Yes, we thoroughly screened donor aphids to ensure that we were only introducing *R. insecticola* and no other microbes. We have added details about the PCR screening methods to the revised paper, including additional methods and a new supplementary table with the primer sequences. We used PCR screens to ensure that *R. insecticola* had established in lines used in our experiment, as described in the revised manuscript (lines 115-121; lines 123-125).**

3) Were there any procedural controls that assessed the effectiveness and uniformity of success of the methods used to infect the aphids with the fungi? I ask particularly because the strongly significant block effect (line 167), when the blocks were treated as fixed variables, suggests not just that repeated blocks differed, but that block 1 differed systematically from block 2. Unless, blocks 1 & 2 differed systematically as part of the experimental design, the fungal infection rates should not have differed strongly and significantly. If that effect is real, it should be presented and explained. Without those clarifications, it is difficult to assess the meaning of Fig. 2.

**We work to keep the infection protocol as consistent across Blocks as possible, but a number of factors could still contribute to differences among blocks. For example, differences in the ambient temperature and humidity of the laboratory can influence entomopathogen infection success. In addition, the dried cadavers we use to maintain fungal stocks in the lab can vary across blocks in terms of number of days in storage, which could influence infection success. By modeling Block as a fixed effect we aim to account for these differences across our experiment. We’ve expanded on this issue in the revised text (lines 150-153, lines 182-184).**

Statistics: I agree that the fungal infection/protection data should be analyzed via a generalized liner model, but:

4) We need a better description of the model used. What type of GLM was used? The methods suggest that a logistic/binomial-family GLM was most appropriate, but the symmetric error bars on Fig. 2 and the use of the ‘multcomp’ package made me suspect that a normal/gaussian GLM was used. With infection rates running as low as 10%, I suspect that the logistic GLM is much more defensible.

**Sorry for the confusion! The reviewer is correct that a logistic/binomial GLM was used for the analysis; we’ve made a new version of Figure 2 with 95% CI (see below) as suggested, which should eliminate any confusion due to the error bars. We’ve clarified these details of the statistical analysis in the revised text (lines 155-156; Figure 2 and caption).**

5) I was surprised that you chose to model the block effect as a fixed effect. Unless Block 1 really did differ systematically from Block 2 in the design of the experiment (e.g., Block 1 was done first and there was a time/duration/extinction effect), it seems more defensible to treat the blocks as random (blocking) effects in a mixed model (e.g., via the ‘glmer’ function in the lme4 package). This is particularly important when comparing the honest-to-goodness fixed effects (here the lines/symbionts) because the blocking effects, in classical theory, become part of the error variance for testing the fixed effects. The ‘glmer’ function handles this properly. - - I think we can ignore the ‘shrinkage’ effects commonly discussed in mixed model random effects because you don’t get much shrinkage with only two blocks with nearly balanced sample sizes.

**Thanks for this comment. Conventional wisdom is that one should not model an effect as random if there are fewer than 5 categories/replicates. Since we included only two experimental blocks, I opted to model block as a fixed effect. But I understand that some disagree with the conventional wisdom (e.g. Gomes 2022, PeerJ). Because keeping block as a fixed effect is arguably the more conservative approach, we’ve opted to keep the model as-is in this revision but are happy to revisit this point if needed.**

6) I have always been nervous about using the ‘multcomp’ package for logistic regressions when some of the effects are close to 0 or 1 (e.g., 0.1 Fig. 2). For GLM’s, multcomp uses a likelihood ratio test that assumes the likelihood is symmetrically distributed around the MLE, which may well not be true in logistic regressions (with some extreme effects) because of the non-linear transformation of the linear result (the logit) to the probability value, and the hard bound on probabilities of infection at 0 and 1. One way to get around this is to use a Bayesian regression and directly test the ‘Bayesian p values’ comparing each line to the ‘None’ control.

**This package has been widely used in the field for post-hoc comparisons with logistic regression. We’ve weakened the language around the claim based on the post-hoc test (the significant increase in fungal susceptibility in aphids harboring *R. insecticola* strain .CF7) – (e.g. line 25, lines 185-186, line 197) . This does not alter our main finding that aphid secondary symbionts do not confer protection against *B. apiculata*.**

Smaller points - Fig. 2 - I was confused by the ‘Percent sporulated’ label on the dependent axis. Isn’t this just percent cadavers (line 137-138)?

**Edited as suggested, see revised Figure 2.**

- The confidence intervals for each line will be asymmetric in a logistic regression, so the ‘+/- 1 SE’ error bars can be misleading. I’d suggest converting to confidence intervals.

**Great suggestion, see the revised version of Figure 2 with 95% CIs.**

Reviewer #3: I read the manuscript by Inchauregui et al. with interest. The paper aims to investigate the impact of the aphid facultative symbiont against the the fungal entomopathogen Batkoa apiculata. In general the topic is relevant however there is some comments:

I suggest authors to motivate the choice of facultative bacteria tested in this study. On the basis of what they were chosen? Please improve this aspect because it is the purpose of the work.

**Thanks; We designed the symbiont panel to reflect a range of phenotypic variation in terms of fungal protection against *P. neoaphidis*, including protective and non-protective strains of *Regiella*, and protective and non-protective species of other symbionts. We included *Regiella insecticola,* in particular, because it is well-studied in terms of protection against *P. neoaphidis* (see Scarborough et al. 2005; Parker *et al.,* 2013, 2017, 2021). Hopefully this is clear from the revised manuscript.**

Section Material and Methods:
Line 109: Please explain how you can verify the infection status of both subclones cured and infected aphid with Regiella insecticola. The infection of the aphid was verified using diagnostic PCR? If yes, please give a full description of the method.

**Please see above, we have added a full description of the PCR methods and a new supplementary table with the primer sequences.**

Line 110: please add your laboratory conditions used in this experiment. Aphids were reared on V. faba seedlings at which temperature? Photoperiod?

**Thanks. Aphids were reared on *V. faba* at 20°C on a 16L:8D photoperiod. We’ve added this information to the revised text.**

Line 113: you used species-specific primers? Give details please? And what about other facultative symbionts such as Hamiltonella defensa? You are sure that your aphid without this bacteria? Have you used specific primers?

**Please see above. Primer sequences are now included in a supplementary table, and there are additional details in the methods (lines 116-121). We are indeed confident that our lines do not harbor *Hamiltonella defensa* or any facultative symbiont other than those indicated.**

Line 128: are you sure for the age of your aphids used in this experiment? 11 days old?!!! I think it’s too old.

**Thanks for this comment. We used this time point (fungal infection at 11 days old) in order to keep this study consistent with several previous studies (e.g. Parker et al. 2021 Molecular Ecology). As the objective was to determine if the protection measured in these previous studies against *Pandora neoaphidis* also extends to *Batkoa apiculata*, it was important to keep the protocol consistent across studies. We clarified the link to previous studies in the revised text (line 140).**

Section Discussion: The discussion section is very short and should be supported with previous work. For example:
1.The aphid facultative symbiont Serratia symbiotica influences the foraging behaviors and the life-history traits of the parasitoid Aphidius ervi (Entomologia Generalis). 2. Effects of pea aphid secondary endosymbionts on aphid resistance and development of the aphid parasitoid Aphidius ervi: A correlative study. (Entomologia Experimentalis et Applicata)

**We appreciate this feedback. We feel that brevity is a feature of this manuscript, and have opted not to expand the discussion, which we feel covers the important findings of our study. We were, in particular, unsure how to include a discussion of the effect of facultative symbionts on other traits like foraging behavior and wasp infection within the context of our study on fungal pathogens, but we are happy to revisit this suggestion if required.**
