# Supplementary material for: Aphid facultative symbionts confer no protection against the fungal entomopathogen Batkoa apiculata
Source: PLoS One. 2023 May 19;18(5):e0286095. doi: 10.1371/journal.pone.0286095 (PMC10198479; doi:10.1371/journal.pone.0286095)
Supplement: Attachment — Submitted filename: answer to authors.docx [file pone.0286095.s005.docx]

This is a manuscript of great quality that deserves to be published in the Plos One. The authors explore novel roles of some common facultative symbionts in the pea aphid. This system has been largely studied, but many reports only focus on positive results. The authors start by screening fungal isolations from the field, and establishing aphid lines hosting one different FS (strains or species) by micro-injection donner blood. This is a classic starting point to go further into the exploration of specific symbiont services. The next step taken by the authors is to study interactions of different aphid lines with a fungal isolate as B. apiculate, and found no protection against the fungal, some even susceptible. This finding adds to the growing body of literature suggesting that the various role of FS. Besides the other reviewers’ suggestion, I have more few minor comments that I am sure the authors can address with no problem.

1) The LSR1-01 pea aphid is generally regarded as only harbor *Buchnera*, but also need screen the nine known FS frequently in the lab. Have you done this before and after the study. If you done, please show all the primers in the Table S1.
2) Besides, it would be a better choice to combine PCR result and FISH microscopy to check the symbiont presence. But here you do not need add the Fish assay.
3) Please here show the ampicillin recipe to readers
4) Line 127, “work to confer no protection against *P. neoaphidis*”. All the strains or only .515. Need more clarify.
5) Line 140 11 days old used in the study. It is a bit old age for aphids. In nature, Fungal are prefer to infect aphid nymphs or adult status. If like to infect nymphs, so why you chosen the 11-d-old.
6) Where is SEM in the Figure 2, and bold your stains in Fig. 1 (if convenient)

Chen Luo

NWAFU- Northwest A&F University- Yangling- China

INRAe - National Research Institute for Agriculture, Food and Environment- Sophia Antiplise- France
 ( I sign all my reviews)
