# Supplementary material for: Aphid facultative symbionts confer no protection against the fungal entomopathogen Batkoa apiculata
Source: PLoS One. 2023 May 19;18(5):e0286095. doi: 10.1371/journal.pone.0286095 (PMC10198479; doi:10.1371/journal.pone.0286095)
Supplement: Attachment — Submitted filename: Response_letter_2.docx [file pone.0286095.s006.docx]

Associate Editor:

The manuscript can be accepted pending minor revision suggested by the reviewers.

- **Thanks for handling this revised submission.**

Reviewer #4

In their paper, Inchauregui et al. studied the influence of facultative symbionts on the ability of aphids to resist a generalist entomopathogenic fungus. They showed that the facultative symbionts tested did not confer any protection against the fungus Batkoa apiculata. Although this is more of a case study (only one species of fungus tested) with negative results that are difficult to generalize, the study is very well done and addresses an area of research that remains understudied. Despite this and its concise form, the study is well written, to the point, and sets the stage for studying the mechanisms behind these protective effects. I have a few minor comments below.

- **Thanks to the reviewer for these comments.**

L48: Perhaps also cite Hansen et al. 2012 (1).

- **Done**

L77: Where do these uninfected aphids exposed to spores come from? From the field? Are these aphids infected by facultative symbionts?

- **Thanks; these are symbiont-free laboratory reared aphids from the LSR1-01 background, we clarified this in the revised manuscript.**

L113-114: Was the screening done before or after the 10 generations were obtained? It is not clear to me if it was done after (which is necessary to ensure that the relationship between host and symbiont has stabilized).

- **Yes the screening was done after the 10 generations. The text reads “…and waited at least 10 generations before using the lines in these experiments, at which point the lines were screened again for symbiotns using PCR.”**

L120: Do you know more about the Serratia symbiotica 509 strain? Are there any studies that have characterized the associated induced phenotypic effects?

- **No, unfortunately this strain has not been used elsewhere in the literature, but multiple other strains of Serratia symbiotica have been studied as cited.**

L128: The aphids are 11 days old. They are young adults. But is this the same developmental stage as in previous studies that showed protective effects against entomopathogenic fungi?

- **Yes, as we specify in the manuscript we used this timepoint to be consistent with previous studies that found the protective effects against fungi.**

L186-187: The subject behind "strains" is not clear. I assume that the authors are referring to Regiella. But since only one strain of Regiella has been tested, the subject should be expressed in the singular.

- **We’ve carefully checked the use of strain vs strains throughout the manuscript and believe this is correct in all cases.**

I have reread the article several times and have no further comments. Perhaps the discussion could have been more in depth (2), but perhaps it is best to keep it concise here due to the brevity of the experience. Perhaps raise the idea that fungal pathogens can switch from parasitism to mutualism as in cicadas with an interest in studying these interactions in other Hemiptera (3–6). I would have many more thoughts to share, but I would stray from the basic article. I hope the authors will teach us more about insect-symbiont-fungus interactions.

- **Thanks!**

Reviewer #6

This is a manuscript of great quality that deserves to be published in the Plos One. The authors explore novel roles of some common facultative symbionts in the pea aphid. This system has been largely studied, but many reports only focus on positive results. The authors start by screening fungal isolations from the field, and establishing aphid lines hosting one different FS (strains or species) by micro-injection donner blood. This is a classic starting point to go further into the exploration of specific symbiont services. The next step taken by the authors is to study interactions of different aphid lines with a fungal isolate as B. apiculate, and found no protection against the fungal, some even susceptible. This finding adds to the growing body of literature suggesting that the various role of FS. Besides the other reviewers’ suggestion, I have more few minor comments that I am sure the authors can address with no problem.

1) The LSR1-01 pea aphid is generally regarded as only harbor *Buchnera*, but also need screen the nine known FS frequently in the lab. Have you done this before and after the study. If you done, please show all the primers in the Table S1.

- **Yes, we do report the primer sequences used for screening in Table S1 and we did screen LSR1 for symbionts. This line was also used in the genome sequencing project and in dozens of subsequent papers, and it does not harbor any other secondary symbionts.**

2) Besides, it would be a better choice to combine PCR result and FISH microscopy to check the symbiont presence. But here you do not need add the Fish assay.

- **Thanks, we’ll keep this in mind as a possibility for future work.**

3) Please here show the ampicillin recipe to readers

- **Thanks, 1% ampicillin was used, this info was added to the revised manuscript.**

4) Line 127, “work to confer no protection against *P. neoaphidis*”. All the strains or only .515. Need more clarify.

- **Thanks, this has been clarified in the revised text.**

5) Line 140 11 days old used in the study. It is a bit old age for aphids. In nature, Fungal are prefer to infect aphid nymphs or adult status. If like to infect nymphs, so why you chosen the 11-d-old.

- **The previous studies demonstrating protection by *Regiella* against *Pandora* were done using 11 day old aphids. We used this same timepoint to keep our study consistent with these previous studies. In collecting fungal isolates for this project and in other studies, we have frequently found adult aphids infected with fungal pathogens; nymphs do not typically get infected, perhaps because of the frequent molting. In either case, 11 day old newly adult aphids is a relevant timepoint for this study.**

6) Where is SEM in the Figure 2, and bold your stains in Fig. 1 (if convenient)

- **We were asked in the last round of review not to include SEM and instead show the 95% confidence intervals in this figure, which is what is shown in the current version of Figure 2.**
